# Improved Efficacy and Reduced Toxicity of Doxorubicin Encapsulated in Sulfatide-Containing Nanoliposome in a Glioma Model

**DOI:** 10.1371/journal.pone.0103736

**Published:** 2014-07-29

**Authors:** Jia Lin, Sarah Shigdar, Ding Zhi Fang, Dognxi Xiang, Ming Q. Wei, Andrew Danks, Lingxue Kong, Lianghong Li, Liang Qiao, Wei Duan

**Affiliations:** 1 School of Medicine, Faculty of Health, Deakin University, Waurn Ponds, Victoria, Australia; 2 Department of Biochemistry and Molecular Biology, West China School of Preclinical and Forensic Medicine, Sichuan University, Chengdu, P. R. China; 3 School of Medical Science and Griffith Health Institute, Griffith University, Gold Coast Campus, Southport, Australia; 4 Department of Surgery, Southern Clinical School, Monash University, Clayton, Victoria, Australia; 5 Institute for Frontier Materials, Deakin University, Waurn Ponds, Victoria, Australia; 6 Liaoning Key Laboratory of Cancer Stem Cell Research, Dalian Medical University, Dalian, China; 7 Storr Liver Unit, Westmead Millennium Institute, the University of Sydney at the Westmead Hospital, Westmead, NSW, Australia; University of Michigan School of Medicine, United States of America

## Abstract

As a glycosphingolipid that can bind to several extracellular matrix proteins, sulfatide has the potential to become an effective targeting agent for tumors overexpressing tenasin-C in their microenvironment. To overcome the dose-limiting toxicity of doxorubicin (DOX), a sulfatide-containing nanoliposome (SCN) encapsulation approach was employed to improve treatment efficacy and reduce side effects of free DOX. This study analysed in vitro characteristics of sulfatide-containing nanoliposomal DOX (SCN-DOX) and assessed its cytotoxicity in vitro, as well as biodistribution, therapeutic efficacy, and systemic toxicity in a human glioblastoma U-118MG xenograft model. SCN-DOX was shown to achieve highest drug to lipid ratio (0.5∶1) and a remarkable in vitro stability. Moreover, DOX encapsulated in SCN was shown to be delivered into the nuclei and displayed prolonged retention over free DOX in U-118MG cells. This simple two-lipid SCN-DOX nanodrug has favourable pharmacokinetic attributes in terms of prolonged circulation time, reduced volume of distribution and enhanced bioavailability in healthy rats. As a result of the improved biodistribution, an enhanced treatment efficacy of SCN-DOX was found in glioma-bearing mice compared to the free drug. Finally, a reduction in the accumulation of DOX in the drug's principal toxicity organs achieved by SCN-DOX led to the diminished systemic toxicity as evident from the plasma biochemical analyses. Thus, SCN has the potential to be an effective and safer nano-carrier for targeted delivery of therapeutic agents to tumors with elevated expression of tenascin-C in their microenvironment.

## Introduction

Indiscriminate exposure of all cells in the body to a systemically administered chemotherapy agent kills healthy cells as well as the tumor cells [1], [2], causing severe toxicity to the patients and leading to serious side effects, and poor quality of life [3], [4]. This non-specific biodistribution and the resulting side-effects limit the clinical application of anticancer drugs [5]. Thus, there is an urgent need to develop new chemotherapeutics that can target tumor cells effectively.

Sulfatide, a lipid that is found in humans, is involved in a variety of biological processes such as cell adhesion, platelet aggregation, cell growth, protein trafficking, signal transduction, neuronal plasticity and cell morphogenesis. Sulfatide is known to bind several extracellular matrix glycoproteins including tenascin-C [6] which is overexpressed in the microenvironment of most solid cancers, including malignant brain tumors [7]. We have recently shown that sulfatide was specifically required for robust uptake of nanoliposomes by human glioblastoma U-87MG cells which overexpress tenascin-C [8], [9]. In addition, in vivo studies demonstrated that the U-87MG tumor-bearing mice received DOX encapsulated in nanoliposomes with sulfatide showed an improvement in survival compared with those received DOX encapsulated in nanoliposomes without sulfatide [8], suggesting that sulfatide in the nanoliposome involves in the binding to tenascin-C. The unique feature of this nanoliposome is that it is comprised of two natural lipids found in human cells, namely sulfatide and 1,2-dioleoyl-*sn*-glycero-3-phosphoethanolamine (DOPE). Thus, this nanoliposome is totally human compatible and degradable. The sulfatide-containing nanoliposomal DOX (SCN-DOX) has been found to remain intact for hours after uptake by the glioblastoma cells. Intracellular distribution study has indicated a high accumulation of DOX in the nuclei where it exerts its cytotoxic effect after 12 h incubation with SCN-DOX at 37°C [8], [10].

Recognising the potential of the use of a tumor environment targeting ligand as one of the main structural constituents of the nanocarriers capable of both passive and active targeting, we designed and carried out a series studies to investigate in vitro and in vivo stability of the SCN-DOX, the pharmacokinetic behaviour, the biodistribution pattern and tumor uptake of the SCN-DOX in a gliomaxenograft model using human glioblastoma cells U-118MG [11]. In the current study, we determined important pharmacokinetic and pharmacodynamic properties of sulfatide-containing nanoliposomal system. First, we studied the stability of the formulation in the presence and absence of serum as well as its in vivo blood clearance. Next, we examined cellular uptake and retention of SCN-DOX in a monolayer culture. Finally, we demonstrated the potential clinical utilities of this nanoliposome via studies on its pharmacokinetics in healthy rats, biodistribution in both healthy rats and tumor-bearing mice as well as antitumor efficacy and toxicity profiles.

## Methods and Materials

### Cell culture

The U-118MG (human glioblastoma) cell line was purchased from American Type Culture Collection (ATCC, Manassas, VA). Dulbecco's modified eagle medium (DMEM, 4.5 g/L D-Glucose), antibiotics and trypsin were from Invitrogen™ (Australia). Fetal bovine serum (FBS)was from Hyclone (Canada). Tissue culture flasks were purchased from BD Falcon™ (Australia). Glass bottom dishes were purchased from MatTek Corporation (Ashland, MA).Cells were cultured in DMEM medium supplemented with 10% fetal bovine serum, penicillin (50 U/mL), and streptomycin (50 µg/mL) in a humidified atmosphere containing 5% CO_2_ and 95% air at 37°C.

### Chromatographic instrumentation and system

Chromatographic instrumentation and system were used based on a previously published method with some modifications [12]. Briefly, the high performance liquid chromatography (HPLC) system used in this study consists of a Waters e2695 Separation Module and a Waters 2475 Multi λ Fluorescence Detector(Waters, USA). The excitation and emission wavelengths were set at the 470 nm and 585 nm, respectively. Chromatographic separation was performed on a Nova-Pak® C18 column (3.9×150 mm i.d., 4 µm, Waters, USA) with a Nova-Pak® C18 guard column (3.9×20 mm i.d., 4 µm, Waters, USA). A mixture of methanol and 0.01 M phosphate buffer (pH = 3.0) was used as the mobile phase. The flow-rate used in the assay was 1 mL/min. The column was maintained at 40±5°C throughout the chromatographic process. All solvents for HPLC procedures were prepared freshly and filtered with 0.22 µm membrane before use.

### Preparation of SCN-DOX

Liposomes were prepared according to a previously published method with modifications [8]. Briefly, as the uptake of SCN in the glioma cells was optimal when the ratio between sulfatide and DOPE was 3∶7 [8], DOPE unilamellar vesicles containing 30% (molar ratio) sulfatide were prepared by a hydration method followed by polycarbonate membrane extrusion. DOPE (13.35 µmol/mL) and sulfatide (6 µmol/mL, Avanti Polar Lipids, Inc.)were dissolved in a mixture of chloroform and methanol (2∶1, v/v), and the lipid mixture, composed of DOPE/sulfatide (3∶7, mol/mol), was transferred to glass tubes. Samples were then reduced to a minimum volume under a nitrogen stream, and stored under vacuum for 24 h at 4°C to completely evaporate the organic solvent. The thin lipid films were hydrated by 1 mL of 250 mM ammonium sulfate (pH 8.5).The samples were placed in an ice-water bath and sonicated under nitrogen for 2.5 min with 50% amplitude using a sonicator (Sonics & Materials, Inc). Following sonication, the liposomes were formed via extrusion through polycarbonate membranes (Avanti Polar Lipids, Inc.) with consecutive pore sizes of 400 nm for 14 times, 200 nm for 14 times and 100 nm for 19 times at room temperature. To establish a trans-bilayer ammonium sulfate gradient, the extruded liposomes were dialyzed against a 250-fold volume of 10% sucrose in 25 mM Trizma at pH 8.5 at 4°C for 24 h. The external buffer was changed 3 times during dialysis. After dialysis of the liposomes, DOX in 10% sucrose at a final concentration of 5 mg/mL was added to the liposomes at a drug-to-lipid ratio of 0.3∶1 (w/w), followed by incubation in the water bath at 60°C for 1 h. Non-encapsulated DOX was removed by size exclusion chromatography using a Sephadex G-50 column.

### Determination of phospholipids in SCN-DOX

The concentration of phospholipids (DOPE) in liposomes was determined as previously described [13]. Briefly, 1 mL chloroform and 0.5 mL ferri-thiocyanate reagent were added in to a 100 µL aliquot of SCN-DOX. The samples were then vortexed for 1 min, and centrifuged at 12,000× *g* for 5 min. Following the removal of supernatant,the absorbance of samples was measured at 488 nm against the chloroform blank. The DOPE concentration in the samples was calculated according to a standard curve of DOPE concentration *vs.* its fluorescence intensity.

### Determination of particle size and zeta potential of SCN-DOX

After the size exclusion chromatography, 10 µL aliquot of liposome was diluted by 990 µL PBS and mixed gently. The vesicle size and zeta potential of SCN were measured using ZetasizerNano ZS Particle Characterization System from Malvern Instruments (Malvern, UK).

### Determination of drug loading efficiency of SCN-DOX

For determination of DOX loading efficiency, standard curves of DOX (ranging from 50 to 10,000 ng/mL) were established via using HPLC initially. Calibration curves were constructed by plotting peak areas of fluorescence derived from DOX vs. DOX concentrations. A linear regression was used for quantitation. The standard formulas were determined by linear regression as y = mx+b, where y is the peak area of DOX and x is the DOX concentration. The DOX concentration in the samples was calculated according to a standard curve of DOX concentration *vs.* its fluorescence intensity.

The amount of DOX encapsulated in SCN was determined by disrupting the liposomes with methanol, followed by quantification of DOX using a fluorescence detector in HPLC. Briefly, 10 µL aliquot of the liposomal drug eluted from a Sephadex G-50 column was diluted in 100-fold phosphate buffer/methanol(45∶55,v/v), and the mixture was centrifuged at 20,000× *g* for 5 min. Then, the supernatant was measured via using HPLC. Encapsulation efficiency was calculated by the following equation:
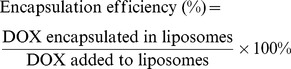



### In vitro release kinetics of SCN-DOX

The in vitro leakage of DOX from SCN was measured by a dialysis method [14], [15]. Briefly, 2.5 mL SCN-DOX was added into a Slide-A-Lyzer Dialysis Cassette (Pierce, molecular weight cut-off of 2 kDa). The dialysis cassette was placed into a beaker containing 250-fold excess of phosphate-buffer saline (PBS) or PBS with 10% fetal bovine serum, penicillin (50 U/mL), and streptomycin (50 µg/mL). The SCN-DOX was dialyzed with stirring for 72 hours at 37°C. At various time points (0 h, 0.5 h, 1 h, 4 h, 8 h, 24 h, 48 h and 72 h), 500 µL aliquots was withdrawn from the external buffer for release kinetics analysis, and replaced with the same volume of fresh external buffer. For HPLC measurement, the aliquots were mixed with 1 mL methanol, followed by centrifugation at 21,000× *g* for 5 minutes. Supernatants were collected for the measurement of HPLC. The drug concentration in the external buffer was calculated according to a standard curve of DOX concentration *vs.* its fluorescence intensity.

### Analysis of cytotoxicity

The viabilities of treated and untreated cells were determined by the MTT (3-(4,5-dimethylthiazol-2-yl)-2,5-diphenyl-2H-tetrazolium bromide) assay which measures the mitochondrial conversion of MTT to formazan as detected by the change of optical density at 570 nm [16], [17]. Briefly, MCF-7 and U-118MG cells were plated at a density of 4×10^3^ and 3×10^3^ cells per well respectively in 100 µl DMEM medium in 96-well plates and allowed to grow for 24 hours. The cells were then exposed to a series of different concentrations of free DOX, SCN-DOX or blank SCN for 48 hours at 37°C under 5% CO_2_. Then, 10 µL MTT solution (5 mg/mL in PBS) was added into each well followed by incubation for 4 h at 37°C. The reaction was terminated by removing MTT before the addition of 150 µL/well solubilisation reagent (dimethyl sulfoxide, DMSO). Absorbance was measured at 570 nm using a VICTOR TM X5 Multilabel HTS Reader (PerkinElmer Life and Analytical Sciences). DOX concentration leading to 50% cell-killing (IC_50_) was calculated using the statistical software package SPSS 13.0.

### Confocal microscopy analysis for cellular uptake and retention of SCN-DOX

U-118MG cells (1.5×10^5^ cells/well) were seeded in 35 mm glass bottom dishes and incubated at 37°C in 5% CO_2_ for 24 h. The medium was then replaced with full culture medium containing 2 µg/mL free DOX or SCN-DOX. After incubation for another 24 hours, cells were washed twice with PBS and incubated with fresh culture medium. The cells were then imaged for cellular uptake studies, followed by serial imaging at 1 h, 2 h, 4 h, and 24 h for retention study using a Fluoview FV10i fluorescence laser scanning confocal microscopy (Olympus, Japan).

### Study of pharmacokinetic properties and biodistribution in vivo

Male Sprague-Dawley (SD) rats (200 to 250 g) were housed in a temperature controlled room (25±1°C) with a 12-h light-dark cycle. Rats were fed *ad libitum* with a standard diet but were fasted overnight before free DOX or SCN-DOX administration. All procedures involving animal experimentations were approved by the Deakin University Animal Welfare Committee.

For determination of DOX concentration in the blood and serum, standard curves of DOX (ranging from 2 to 10, 000 ng/mL) were established via using HPLC initially. The construction of calibration curves and the determination of standard formulas were the same as described above. The DOX concentration in the samples was calculated according to a standard curve of DOX concentration *vs.* its fluorescence intensity.

To investigate the pharmacokinetics (PK) properties and biodistribution (BD) of SCN-DOX in vivo, healthy SD rats were injected i.v. with free DOX or SCN-DOX via the tail vein with a single dose of 5 mg DOX/kg. For PK study, 250 µL blood was serially collected from the same animal in heparinised tubes from the tail at 2 min, 0.5 h, 2 h, 6 h, 24 h and 48 h. After collection, samples were centrifuged at 3,000× *g* at 4°C for 10 min to separate the plasma. To determine DOX levels in plasma, 495 µL of methanol and 405 µL of phosphate buffer were added into 100 µL plasma, vortexed for 1 min, and centrifuged at 21,000× *g* for 10 min at 4°C. The supernatant was transferred to another tube followed by the addition of 2 µL of perchloric acid (35%, v/v). The samples were vortexed for 1 min, and centrifuged at 21,000× *g* for 10 min at 4°C, followed by the measurement of DOX concentration using HPLC [18]. For biodistribution study in rats, animals were sacrificed by Lethabarb R (100 mg/kg) at 0.5 h, 2 h, 4 h and 24 h after a single dose of DOX or SCN-DOX injection. The biodistribution study in tumor-bearing mice was performed after the administration of 6 doses of DOX or SCN-DOX. Tissues (heart, liver, spleen, lung and kidney) were collected, followed by washing in cold physiological saline to remove any excess blood, blot-dried using filter paper and weighted. One hundred µg of tissue was added to 495 µL of methanol and 405 µL of phosphate buffer in a tightly sealed 2-mL tube followed by homogenization using the FastPrep®-24 tissue and cell homogenizer (MP Biomedicals, US). The tissue homogenate was centrifuged at 21,000× *g* for 10 min at 4°C and the supernatant was transferred to a fresh tube and extracted with the addition of 2 µL perchloric acid (35%, v/v) as described above. The supernatant was analysed using HPLC [19].

### Tumor implantation, treatment and evaluation in U-118MG tumor-bearing mice

Six-week-old female BALB/c-Foxn1^nu^ mice were purchased from The Animal Resources Centre (Perth, Australia) and used for U-118MG xenograft models. The mice were housed in a TECNIPLAST Sealsafe™ Individually Ventilated Cages which were placed in the temperature controlled room (25±1°C) with a 12-h light-dark cycle. Mice were fed *ad libitum* with a standard diet. All procedures, involving animal experimentations, were approved by the Deakin University Animal Welfare Committee (AEC Project Number: A61-2008 and A62-2008).Beddings, cages and water were autoclaved at 121°C for 30 minutes while the fodder was sterilized by ultraviolet irradiation before use. For tumor implantation, a U-118MG single cell suspension was prepared by trypsinization followed by washing and resuspension in PBS at a concentration of 5×10^7^ cells/mL. Half million of cells in 100 µL PBS were inoculated subcutaneously (s. c.) to the right flank of the mice. Tumor diameters were measured using a digital calliper every other day after implantation and approximate tumor volume was calculated as length×width^2^/2 (*V* = *lw* ^2^/2). The tumor was allowed to grow until its volume reached approximately 150 mm^3^ [20].

Once the tumor reached a volume of 150 mm^3^, the nude mice were randomly divided into 2 experimental groups (5 to 6 mice per group). Formulations of either free or liposomal DOX at doses of 5 mg/kg DOX or equivalent were administered once a week via the tail vein at rate 0.4 mL/min for 6 weeks. Twenty-four hours after the last injection, mice were sacrificed by injection of Lethabarb R (100 mg/kg). Tumors and tissues were collected and processed as described in section 2.8 [20], [21].

### Therapeutic efficacy in tumor xenograft model

For therapeutic studies, the nude mice were randomly divided into 4 experimental groups (5 to 6 mice per group) when the xenograft tumors reached 150 mm^3^ [22]. Treatment groups consisted of (a) saline control group, (b) free DOX (5 mg/kg) group and (c) SCN-DOX (5 mg/kg in DOX), (d) blank SCN group. The treatment was administered by intravenous (i. v.) injection via the tail vein once a week for six weeks [23]. Tumor sizes, as well as animal weights, were measured every other day.

### Determination of biodistribution and systemic toxicity in mice

For tissue distribution after repeated injections, mice were i.v. injected with 5 mg/kg free DOX or SCN-DOX (5 mg/kg in DOX) once a week for 6 weeks and were sacrificed 24 hours after the last injection. Tumors and tissues were collected for HPLC measurement of DOX. To evaluate the general toxicity of free DOX and SCN-DOX, 20 female mice (6 weeks old) were randomly divided into 4 groups, and treated as describe above. The mice were sacrificed at 72 days after the 6th injection. Plasma biochemical analyses were carried out by a veterinary pathology laboratory (Gribbles Veterinary Pathology, Clayton, Vic). For the measurement of the serum troponin, the method employed a cut-off threshold of <0.01 µg/L for normal subjects [24]. The concentration of troponin for samples <0.01 µg/L could not be reliably determined nor reported by the veterinary pathology laboratory.

### Data analysis

Results were reported as mean and standard error (mean ± S.E.) unless otherwise stated. The pharmacokinetic parameters were calculated from the average plasma concentrations using the pharmacokinetic software DAS 2.0 software (Mathematical Pharmacology Professional Committee of China, Shanghai, China). The differences in the mean values among different groups were analysed using a one-way analysis of variance (ANOVA) using the statistical software package SPSS 13.0. *P* values less than 0.05 were considered to be statistically significant.

## Results

### Characterization of liposomes

The physicochemical characteristics of SCN are presented in Table 1. Mean vesicle size of SCN incorporating DOX was 92.32±1.31 nm with polydispersity index (PDI) of 0.15±0.01. At an initial input weight ratio of DOX to DOPE of 0.3∶1, the efficiency of DOX loading to SCN using ammonium sulfate gradient was 94.11%±2.27%, in consistence with what is reported in the literature [25]. Zeta potential value of SCN was −26.38±2.20 mV. The DOX to DOPE weight ratio after DOX encapsulation into SCN was determined to be 0.5∶1.

**Table 1:** Physical properties of the liposomal formulations.

Composition	DOX loading efficiency (%)	Particle size (nm)	DOX-to-DOPE ratio (w/w)	polydispersity index (PDI)	Zeta potential (mV)
SCN	94.11±2.27	92.32±1.31	0.5∶1	0.15±0.01	−26.38±2.20

Data are shown as means ± S.E. of at least three independent experiments.

### In vitro drug retention properties

**Figure 1:**
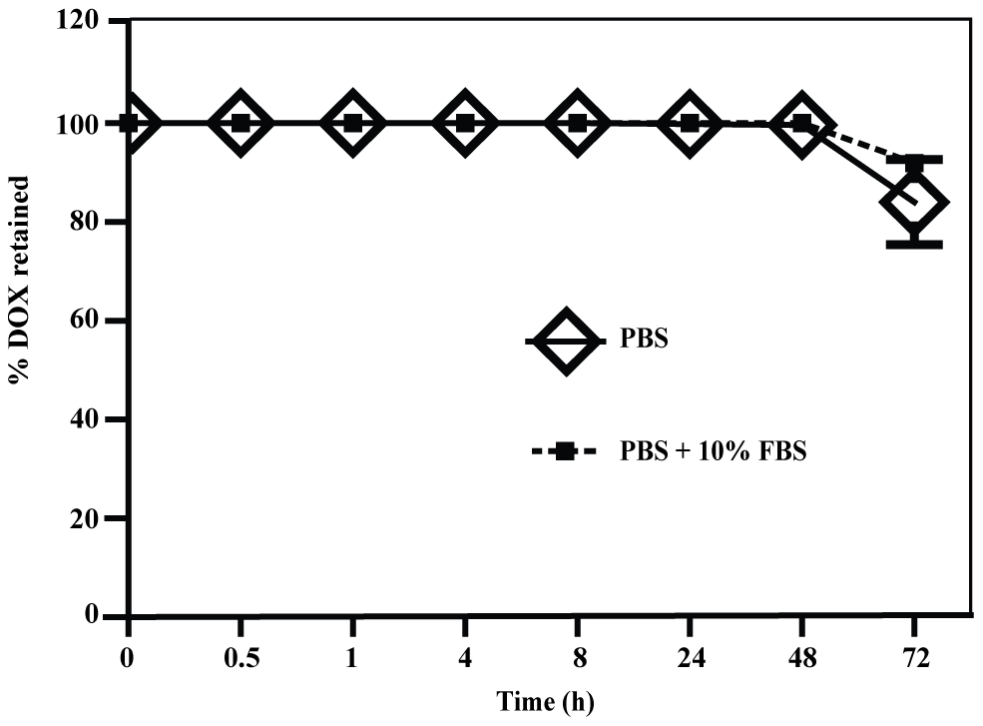
In vitro stability of SCN-DOX. The stability of SCN-DOX was studied by dialyzing the release of DOX from SCN-DOX into PBS or PBS with 10% FBS at 37°C. Aliquots of dialysis buffer were collected at designed time points (0, 0.5, 1, 4, 8, 24, 48 and 72 hour). DOX released into the dialysis buffer was quantified. Data are shown as means ± S.E. of at least three independent experiments.

In vitro DOX release from SCN was determined by dialysing SCN-DOX against PBS or PBS with 10% FBS at 37°C and measuring the DOX concentration over time from the fluid within the dialysis container. As shown in Figure 1, there was minimal DOX leakage from the SCN during the first 48 h dialysis period, with more than 99% of DOX retained in the SCN after 48 h under both PBS and PBS/serum dialysis conditions. The release of DOX increased after 48 h incubation. The percentage of DOX retained in the SCN after 72 h were 84.06%±8.63% in PBS and 91.91%±1.36% in PBS with 10% serum, respectively.

### In vitro cytotoxicity

The cytotoxicity of free DOX and SCN-DOX to U-118MG glioblastoma cells was compared. A breast cancer cell line, MCF-7, that does not express detectable levels of tenascin-C [26] was used as a negative control to verify the role of sulfatide in the interaction between SCN and tenascin-expressing cancer cells. The IC_50_ values for DOX and SCN-DOX are shown in Table 2. The IC_50_ for free DOX (2.51±0.33 µg/mL) in U-118MG was lower than that observed in U-118MG treated with SCN-DOX (19.55±0.68 µg/mL). These results suggested that U-118MG cells were relatively more sensitive to free DOX than to SCN-DOX when exposed to a constant concentration of the agent. There was no significant toxicity of blank SCN after 48 h incubation (data not show). The lower toxicity of SCN-DOX to U-118MG in vitro is in a good agreement with the literature for other liposomes loaded with free DOX [27], [28] and is consistent with the above stability study as the cytotoxicity in vitro is related to the release rate of DOX from nanocarriers [15]. The data suggest that under in vitro conditions, the cellular uptake of free DOX via random diffusion is more efficient than that of SCN-DOX via energy-dependent endocytosis. Moreover, the IC_50_ value of SCN-DOX in MCF-7 cells was significantly higher than that in U-118MG cells (29.58±2.55 µg/mL and 19.55±0.68 µg/mL, respectively), which might result from differential binding of SCN to cells with or without tenascin-C and resultant difference in the subsequent uptake of SCN-DOX.

**Table 2:** Mean IC_50_ values (µg/mL of doxorubicin) for treatment with free dox and sulfatide-containing nanoliposomal Dox.

Cell line	Free DOX (µg/ml)	SCN-DOX (µg/ml)
U-118MG	2.51±0.33	19.55±0.68
MCF-7	1.76±0.41	29.58±2.55

Data are shown as means ± S.E. of three independent experiment performed in triplicate.

### Intracellular uptake and retention of SCN-DOX in U-118MG cells

**Figure 2:**
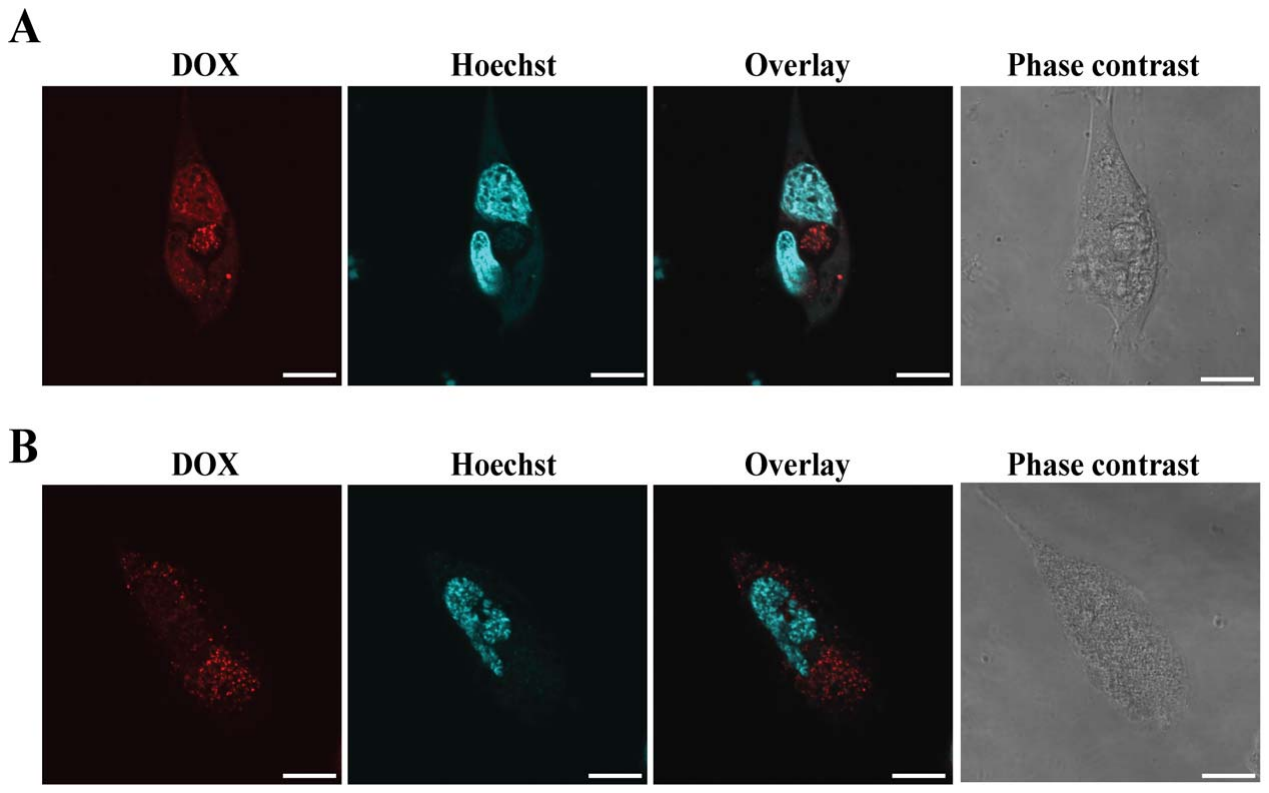
Intracellular uptake of SCN-DOX in U-118MG cells. U-118MG cells were incubated with 2 µg/mL free DOX or equivalent SCN-DOX for 24 hours. Cells were washed twice with PBS and imaged with a confocal fluorescence microscope. (**A**) Cells treated with free DOX. (**B**) Cells treated with SLC-DOX. Red: fluorescence from DOX; blue: nuclei stained with Hoechst 33342. Data are representative of three independent experiments. Scale bars: 20 µm.

**Figure 3:**
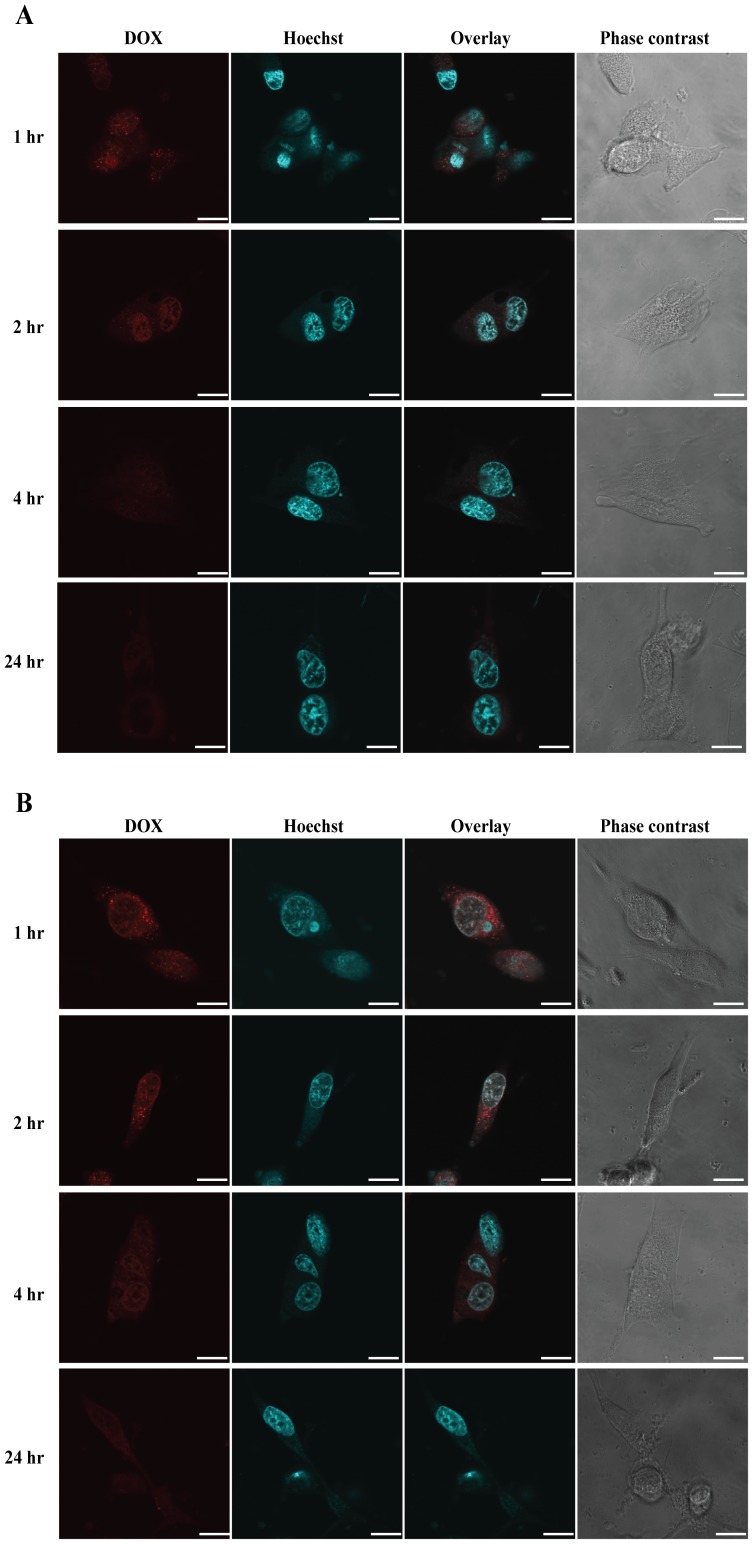
Intracellular retention of SCN-DOX in U-118MG cells. U-118MG cells were first incubated with 2 µg/mL free DOX or equivalent SCN-DOX for 24 hours. Cells were then washed with PBS twice and cultured in fresh full culture medium. The same wells of cells were imaged serially at 1 h, 2 h, 4 h and 24 h after washing using fluorescence confocal microscopy. (A) Cells treated with free DOX. (B) Cells treated with SLC-DOX. Data are typical of three independent experiments. Red: fluorescence from DOX; blue: nuclei stained with Hoechst 33342. Scale bars: 20 µm.

The accumulation and retention of DOX and SCN-DOX in U-118MG cells were studied using laser scanning confocal microscopy utilising the natural fluorescent property of DOX. We first established that there was no apparent gross adverse effects in the cells after 24 h incubation with 2 µg/ml DOX or equivalent amounts of SCN-DOX followed by washing with PBS. Next, cellular uptake of free DOX or SCN-DOX by U-118MG in DOX- or liposome-free medium was examined. As shown in Figure 2, both free DOX and SCN-DOX accumulated in the cytoplasm of glioblastoma cells. However, there was slightly stronger DOX fluorescence (red) in cells treated with free DOX when compared to those treated with SCN-DOX after 24 h incubation. The overlay of Hoechst staining (nucleus) and red fluorescence (DOX) shown in Figure 3 indicated that SCN-DOX were not adhered on the cell surface but actually penetrated into the nucleus. Interestingly, SCN-DOX was better retained by the glioma cells. As shown in Figure 3A, there was a significant decrease of DOX fluorescence in cells treated with free DOX only 2 h after washing with PBS. In contrast, DOX fluorescence could be found in glioma cells treated with SCN-DOX even 24 h after washing (Figure 3B), suggesting more sustained retention of DOX in U-118MG cells when delivered via SCN-DOX than that by free DOX. The improved retention of DOX encapsulated with SCN in vitro implies the potential of better treatment efficacy of SCN-DOX in vivo.

### Improved pharmacokinetic properties of SCN in healthy rats

The pharmacokinetic properties of both free DOX and SCN-DOX were studied in healthy male SD rats. The main pharmacokinetic parameters are shown in Table 3. In our study, the terminal half-life with free DOX in plasma was 20.65±1.34 h, which is consistent with data from earlier studies by others [29], [30]. Whereas it was 41.89±3.58 h with SCN-DOX, showing a2.03-fold increase in the terminal half-life. Indeed, the steady state volume of distribution (Vd) of free DOX (18.36 L/kg) was 25-fold higher than SCN-DOX (0.73 L/kg), suggesting that circulating SCN-DOX sequester drug in the plasma compartment, contributing to the high Cmax observed for SCN-DOX. The area under the plasma concentration-time curves (AUC_0-∞_) of DOX delivered through SCN was 2.06-fold higher than free DOX (3597.03±99.36 µg/Lh and 1746.87±69.94 µg/Lh, respectively), suggesting a decreased in non-specific binding as well as a selective sequestration of the drug to tissues when administered as entrapped in SCN, leading to enhanced bioavailability.

**Table 3:** Pharmacokinetic parameters for free DOX and SCN-DOX.

Formulations	AUC_0-∞_(µg/Lh)	t_1/2_(h)	V_d_(L/kg)
Free DOX	1746.87±69.94	20.65±1.34	18.36±0.80
SCN-DOX	3597.03±99.36**	41.89±3.58**	0.73±0.01**

Data are shown as means ± S.E. of at least three independent experiments.

AUC: Area under the plasma concentration-time curves.

t_1/2_, Elimination half-life.

V_d_: Volume of distribution.

**, *P*<0.01 compared to free DOX.

### Tissue distribution advantages of SCN-DOX in healthy rats

**Figure 4:**
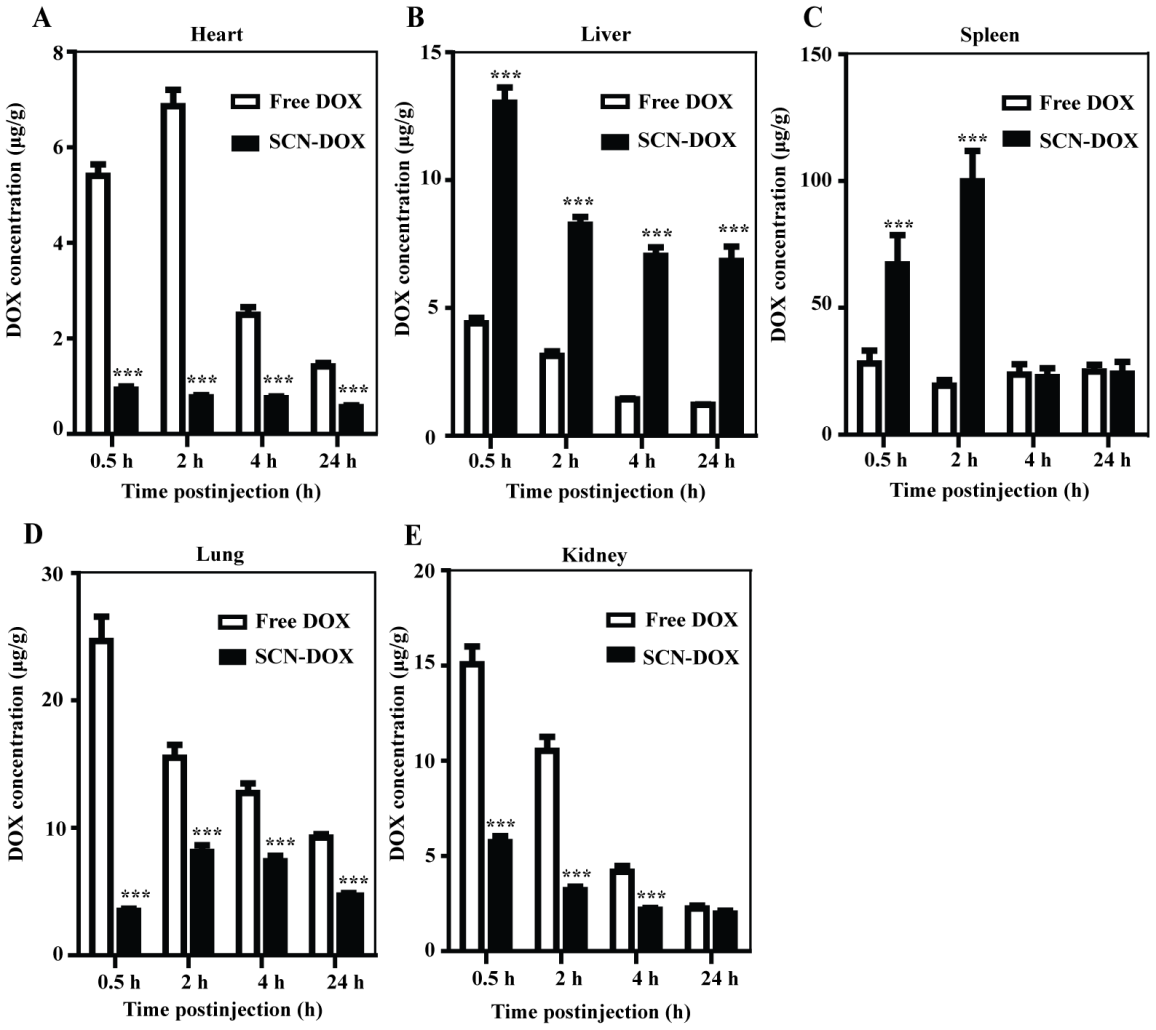
Biodistribution of DOX encapsulated in SCN in SD rats. Healthy rats were injected with a single dose of with 5/kg free DOX or SCN-DOX i. v. Rats were euthanized at different time points. Organs were harvested, washed, weighed, and the DOX was extracted and quantified. Data are shown as means ± S.E. for µg DOX per g of tissue (n = 5–6). ***P<0.001 compared to free DOX.

We next evaluated the biodistribution characteristics of SCN-DOX after i. v. injection of a single dose of 5 mg/kg free DOX or SCN-DOX in healthy SD rats. As shown in Figure 4, treating the rats with SCN-DOX led to significantly decreased DOX accumulation in the heart, the lung and the kidney. In the heart and the lung, the DOX concentration was significantly lower in the animals received SCN-DOX than those receiving free DOX at all time points, typified by a 9-fold and 2-fold lower concentration in the SCN-DOX group at 2-h for the heart and lung, respectively (Figure 4A and 4D). Moreover, distribution of DOX at 0.5 h, 2 h and 4 h time points in the kidney was significantly reduced by i. v. administered SCN-DOX when compared to the free DOX (Figure 4E). Consistent with the reported enhanced sequestration of nanoparticles, including liposomes, by organs of the reticuloendothelial system [29], [31], the DOX concentration was significantly higher in the liver in animals received SCN-DOX than those received free DOX (Figure 4B). In the spleen, the DOX concentration in the SCN group was significantly higher than the free DOX group only initially during the first 2 h after the administration. There was no statistically significant difference of the DOX concentration at 4 h and 24 h points in the spleen between different treatment groups (Figure 4C).

### Improved tumor uptake and biodistribution in U-118MG tumor xenograft model

**Figure 5:**
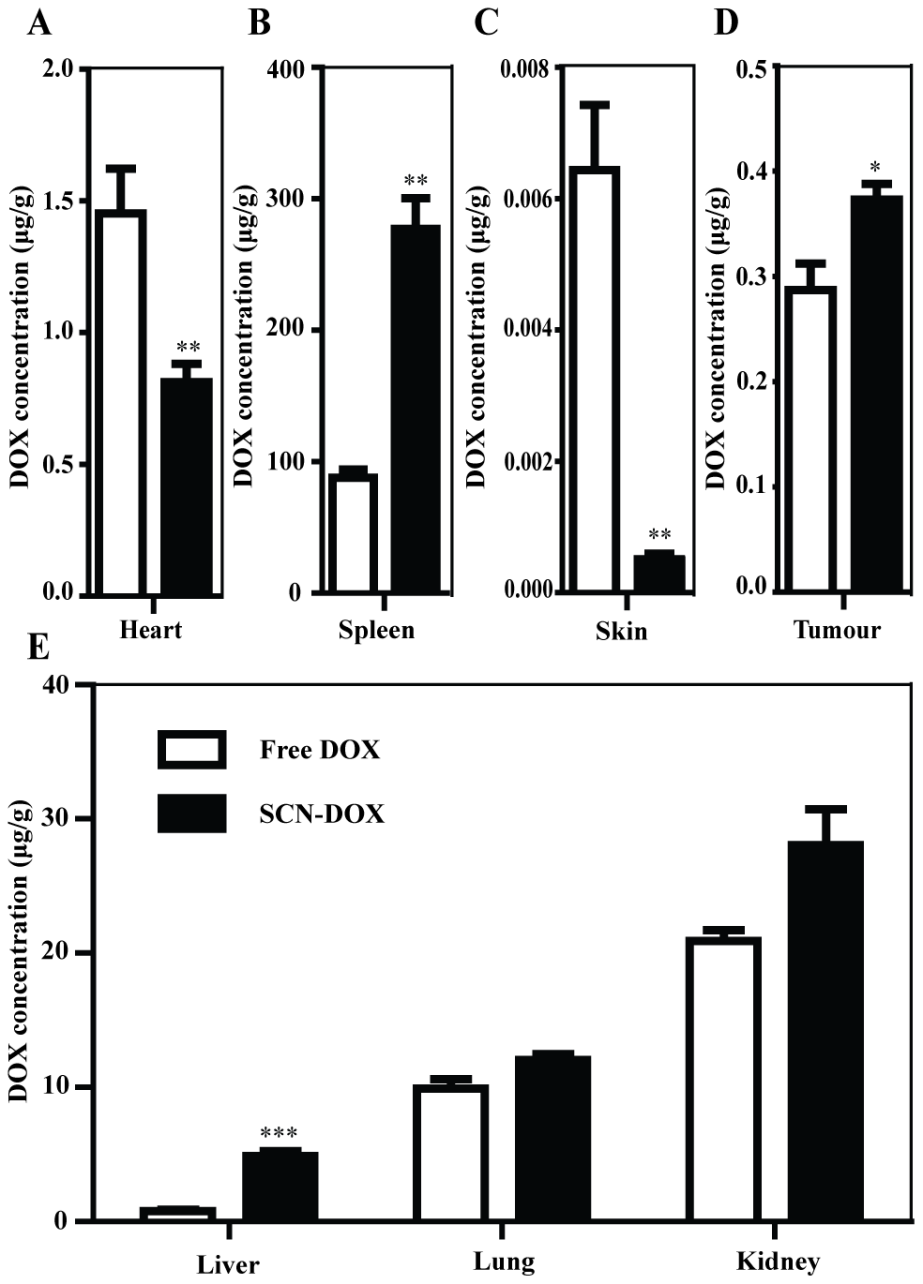
Biodistribution and tumor uptake of DOX encapsulated in SCN in glioma-bearing mice. Nude mice bearing human glioblastomaU-118MGxenografts (s.c.) were treated with 5 mg/kg free DOX or SCN-DOX i.v. once a week for 6 weeks. Mice were euthanized 24 hours after the last treatment. Organs and tissues were harvested, washed, weighed, and the DOX content in tissues, expressed as µg DOX per g tissue, was determined. Data are shown as means ± S.E. (n = 5–6). *, *P*<0.05 compared to free DOX; **, *P*<0.01 compared to free DOX; ***, *P*<0.001 compared to free DOX.

Next, we investigated the ability of SCN-DOX to enhance the delivery of therapeutic agents to tumor in vivo using a mouse tumor xenograft model. Mice bearing U-118MG tumors were administrated intravenously with5 mg/kg free DOX or SCN-DOX once in a week for 6 weeks when subcutaneous implantation tumors reached a volume of 150 mm^3^.The uptake of DOX in various organs and tumor was determined 24 h after the last injection. Consistent with the results from the study in healthy SD rats in the preceding section, the DOX concentration in organ responsible for dose-limiting toxicity in clinics, i.e. the heart, in the tumor-bearing mice treated with SCN-DOX was significantly lower than those treated with free DOX (0.81±0.07 µg/g versus 1.45±0.17 µg/g) (Figure 5A). The DOX concentration in the other known major DOX toxicity organ, the skin, was also statistically significantly lower in the SCN-DOX group compared to the free DOX animals (∼15 fold lower), so as in the kidneys (28.05±2.67 versus 20.91±0.82 µg/g) (Figure 5C and 5E). On the other hand, there were significantly higher levels of DOX in the liver, spleen and lung in the groups treated with SCN-DOX when compared to the free DOX group (6.10-fold, 3.16-fold and 1.22-fold, respectively) (Figure 5B and 5E).However, at least in the case of the liver, the increased accumulation of DOX via SCN-DOX delivery did not seem to translate into enhanced hepatic toxicity (see below).As for the xenograft glioma (Figure 5D), there was a statistically significant elevation of DOX level in tumor tissue in the SCN-DOX group compared to that of free DOX (1.30-fold), confirming the enhanced intratumoral DOX delivery by SCN-DOX in vivo.

### Enhanced therapeutic efficacy of SCN-DOX in U-118MG xenograft tumor model

**Figure 6:**
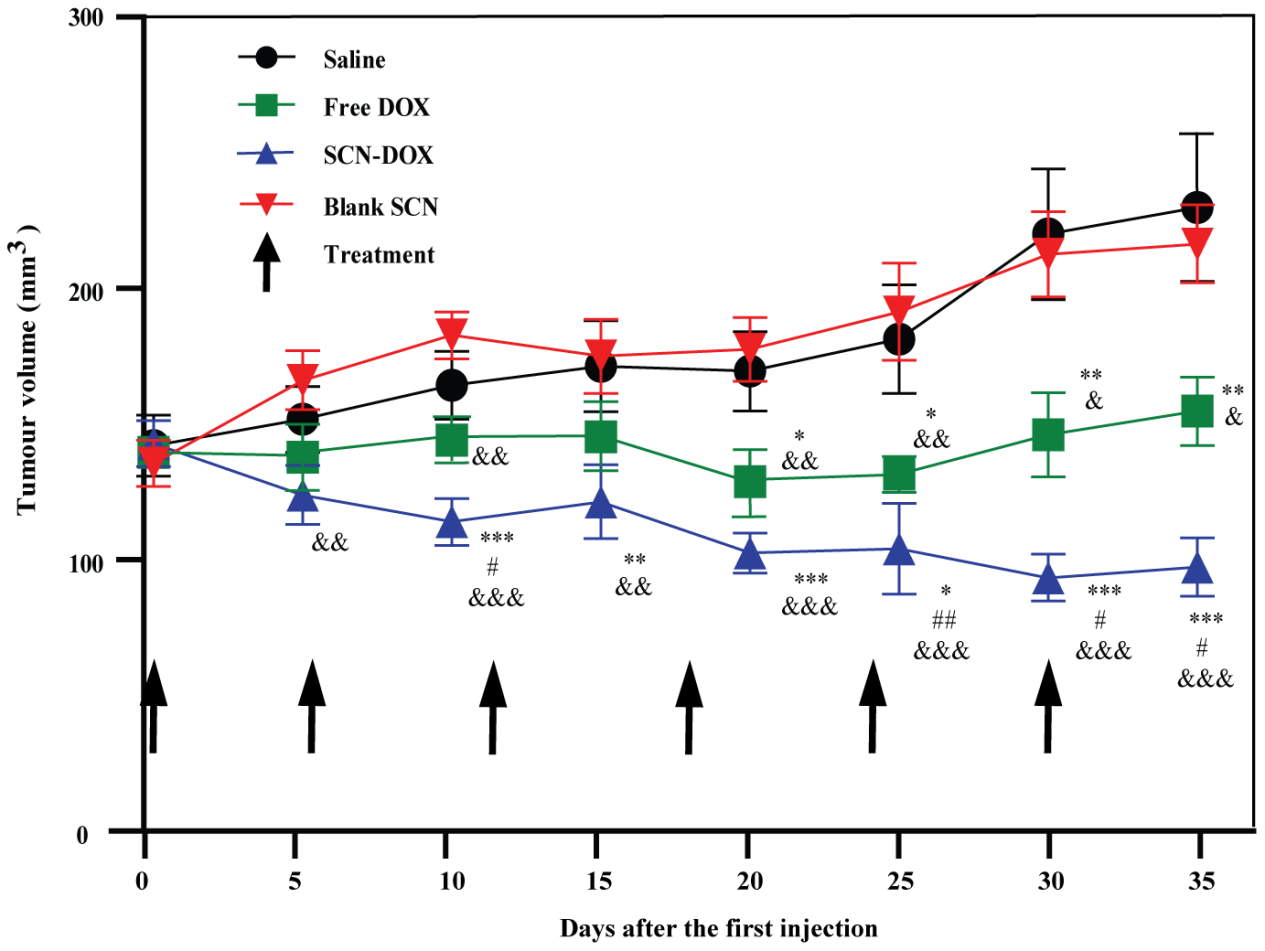
Improved therapeutic activity of SCN-DOX against gliomaxenograft. Mice bearing U-118MG xenografts were injected i.v. with saline, 5 mg/kg of free DOX, SCN-DOX or empty SCN once a week for 6 weeks when tumor volume reached approximately 150 mm^3^. Data shown are means ± S.E. (n = 5–6). *, *P*<0.05 compared to saline; **, *P*<0.01 compared to saline; #, *P*<0.05 compared to free DOX; ***, *P*<0.001 compared to free DOX; ##, *P*<0.05 compared to free DOX; &, *P*<0.01 compared to blank SCN; &&, *P*<0.01 compared to blank SCN; &&&, *P*<0.001 compared to blank SCN.

Given our SCN-DOX is able to deliver more therapeutic agents to the xenograft tumor (Figure 5), we proceeded to determine the antitumor activity of SCN-DOX in vivo. Mice bearing U-118MG tumors were injected with saline, blank SCN, DOX in solution or encapsulated within SCN once in a week for 6 weeks when subcutaneous implantation tumors reached a volume of 150 mm^3^. As shown in Figure 6, there was no significant difference of tumor sizes between mice treated with saline control and blank SCN during the study period. At dose of 5 mg/kg, both DOX formulations were effective in suppressing tumor growth compared to saline and blank liposome control after the 2nd injection. Importantly, tumors grew more rapidly in the mice receiving free DOX when compared with those receiving SCN-DOX. The final mean tumor load was 97.29±10.71 mm^3^ in SCN-DOX treatment group while in free DOX group was 154.76±12.53 mm^3^. Thus, SCN-DOX formulations displayed stronger tumor growth suppression than free DOX.

**Figure 7:**
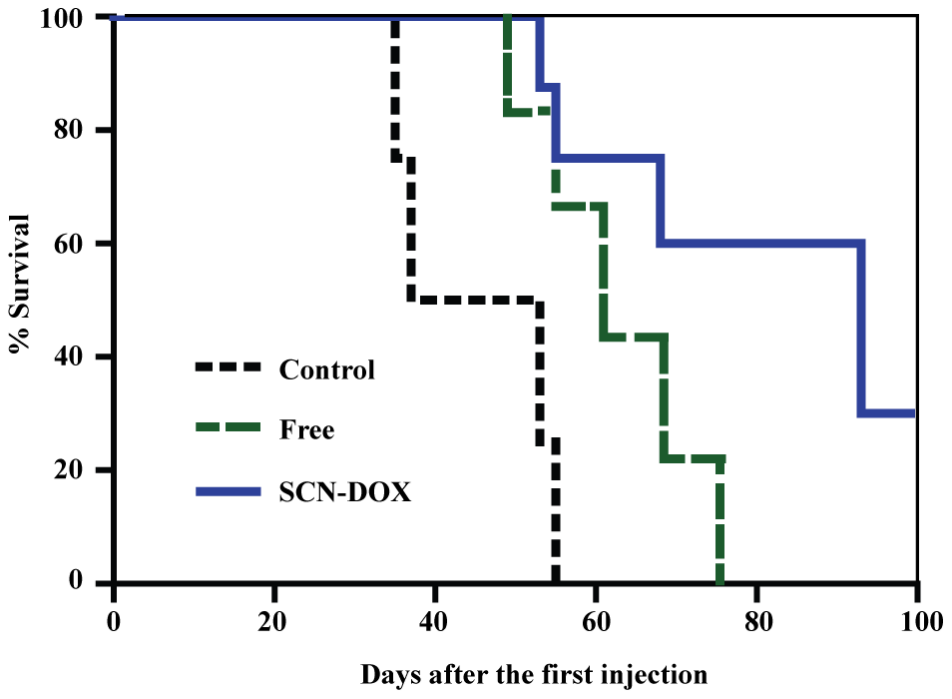
SCN-DOX enhanced survival of tumor-bearing mice. The Kaplan-Meier survival curve shows improvement of life span of U-118MG xenograft-bearing mice treated with SCN-DOX (n = 8–10 per group). Mice were treated as indicated in the legend for Fig. 6 and were sacrificed when the body weight loss was more than 15%.

To further confirm the antitumor efficacy, we compared the survival rates of tumor-bearing mice after different treatment regimen. As shown in Figure 7, the median survival days for saline, free DOX and SCN-DOX group were 45, 61 and 93 days, respectively. Thereby, the medium life-span was increased in mice treated with SCN-DOX by 2.07-fold and 1.52-fold compared to those treated with saline or free DOX, respectively. Taken together, i. v. administration of SCN-DOX for 6 times over a 6-week period displayed not only the stronger suppression on tumor growth but also much improved survival of U-118MG xenograft-bearing mice.

### Reduced toxicity of SCN-DOX in vivo

**Figure 8:**
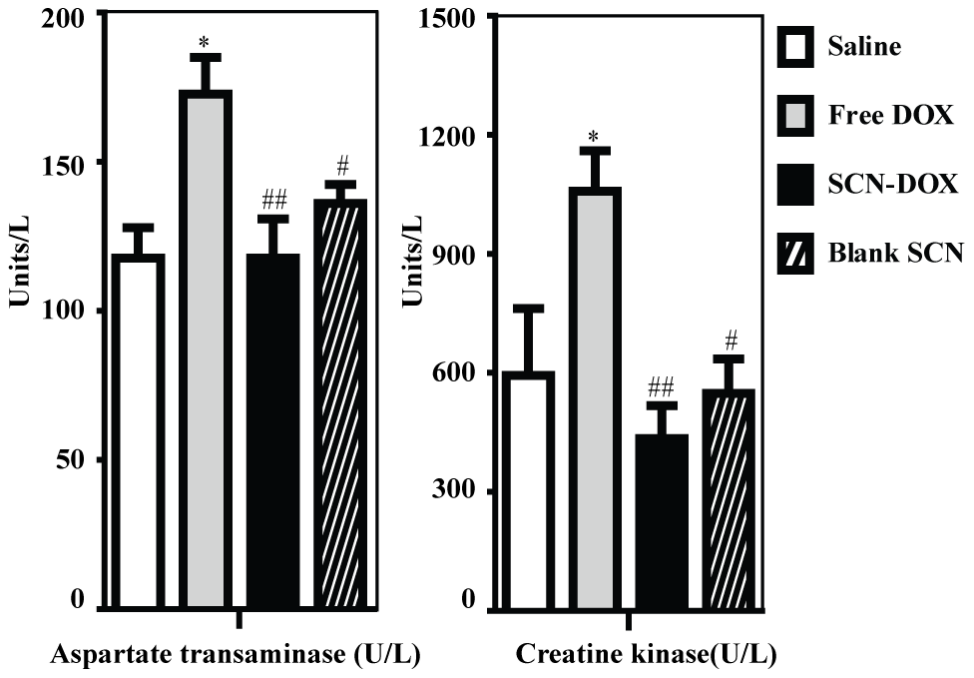
SCN-DOX treatment had significantly reduced cardiac and hepatic toxicity. U-118MG xenograft-bearing mice were treated as indicated in the legend for Fig. 6. Blood was collected immediately after the mice were sacrificed upon reaching the end point. Serum enzymes indicative of cardiac and hepatic toxicity were analysed. Data shown are means ± S.E. (n = 3–5). *, *P*<0.05 compared to saline; #, *P*<0.05 compared to free DOX; ##, *P*<0.01 compared to free DOX.

Clinically, the efficacy of DOX is limited by dose-limiting toxicities. One objective of delivery of chemotherapy agents in a nano-formulaiton is to reduce systemic toxicities. For this aim, we evaluated the toxicity of SCN-DOX and free DOX after repeated injection in tumor-bearing nude mice. The tissue concentrations of DOX were measured 24 h after the 6th administration of either SCN-DOX or free DOX. As shown in Figure 5A and 5C, consistent with our findings with healthy rats, the concentrations of DOX in the heart and skin were significantly lower, ∼1.8-fold and ∼15-fold, respectively, in the tumor-bearing mice treated with SCN-DOX than those treated with free DOX after repeated administrations. As for plasma biochemistry analysis 72 days after the last injection (Figure 8), treatment with free DOX induced a significant increase in serum creatine kinase (CK) concentration (1048.00±100.95 U/L), indicative of heart damage, that exceeded saline control (588.50±167.37 U/L), blank SCN (543.40±86.47) and SCN-DOX groups (430.60±82.94 U/L). Furthermore, compared with the free DOX group, the plasma concentrations of aspartate transaminase (AST), a sensitive indicator of liver damage, in the SCN-DOX group (*p*<0.01) and blank SCN group were significantly lower (*p*<0.05). Moreover, as a marker of DOX-damaged myocytes, troponin was measured for the evaluation of DOX-induced cardiomyopathy in vivo [32], [33]. The method used in the present study has a cut-off threshold of <0.01 µg/L for normal subjects [24]. As shown in Table 4, there was a 1.8-fold higher level of serum troponin in the mice treated with free DOX than those with saline control. In contrast, there is no elevation of serum troponin level in the mice treated with SCN-DOX or blank SCN compared to the controls. The results indicated that the SCN-DOX has the potential to minimize the cardiotoxicity of free DOX.

**Table 4:** Plasma levels of troponin.

Treatment	Number of nude mice	Troponin (µg/L)
Saline	4	<0.01
Free DOX	5	0.018±0.003
SCN-DOX	5	<0.01
Blank SCN	5	<0.01

Data are presented as means ± S.E. (n = 4–5).

## Discussion

As liposomes are composed of naturally biodegradable substances, they are metabolized and cleared while in circulation or upon reaching the target sites, making them safe novel drug delivery carriers [34]. Recently, we have shown that SCN-DOX displays favourable pharmaceutical properties in a colorectal cancer (HT-29) xenograft model, with an improved biodistribution, enhanced treatment efficacy and diminished toxicity compared with the free DOX [35]. Here, we provided the first report on detailed pharmacokinetic study, biodistribution in healthy animals as well as in tumor-bearing mice and antitumor efficacy in a glioma xenograft model [36] to demonstrate that the efficacy of this novel nanoliposome is not restricted to gastrointestinal tumors.

In the literature, most of the reported DOX-to-lipid ratio after loading was between 0.2∶1 and 0.3∶1 (w/w) [37], [38], [39]. In this study, we have achieved a DOX-to-lipid ratio of 0.5∶1 (w/w) for SCN after encapsulation (Table 1). Therefore, our SCN may have the potential to encapsulate more drugs than some of the other types of nanoliposomes. Stable encapsulation is essential for efficient drug delivery to the target site. Our in vitro stability study suggest that SCN are very stable for 48 h 37°C and retain substantial physical stability at 72 h in vitro (Figure 1). Interestingly, the percentage of liposomal DOX leakage after 24 h incubation in other studies is generally more than 5% [15], [40], [41], [42], in contrast to the minimal payload leakage of SCN-DOX at 48 h reported here. Therefore, SCN display superior DOX retention property in vitro under the experimental conditions used. The prolonged half-life of our SCN-DOX in vivo (Table 3) might have resulted from the slow release of DOX from the nanoliposomal formulation [43].

As for in vitro cytotoxicity between different liposomes and cancer cell lines, published studies reported contradictory results. For example, in MLLB2 cells [44] and MCF-7/ADR cells [45], the IC_50_ of the liposomal formulation was significantly lower than free DOX, which indicated a higher cytotoxicity of liposomal DOX in vitro. On the contrary, in HepG2 cells [20] and U-87 cells [8], free DOX seems to have higher intracellular uptake with associated higher cytotoxicity than that of liposomal DOX. Obviously, the kinetic properties are different between liposomal DOX and free DOX in vivo. The half-life of liposomal DOX can be up to several days while the free DOX can be eliminated in few minutes [46], [47], [48]. Moreover, the MTT assay used to derive the IC_50_ is carried with the monolayers in culture dishes, which are very different when compared to the 3-dimentional tissue architecture in vivo [49]. Thus, comparison of IC_50_ in vitro, which is only relevant to the cytotoxicity under a consistent drug concentration, is not a reliable predictor of the therapeutic efficacy in vivo [50]. Furthermore, the SCN-DOX had been found to remain in the nuclei for several hours even after washing under in vitro condition (Figure 3). This is in agreement with other study using the same DOX delivery system in a different cell line [8]. Notably, despite the higher IC_50_ value of SCN-DOX in vitro, it had much better anti-tumor efficacy over the free DOX in U-118MG tumor-bearing nude mice (Figure 6). Interestingly, IC_50_ for free DOX in MCF-7 cells was lower than that for U-118MG, however, the IC_50_ for SCN-DOX was 1.5-fold higher in MCF-7 cells than that in U-118MG cells (Table 2). The higher toxicity of SCN-DOX in U-118MG which overexpress tenascin-C might be, at least in part, attributed to the interaction between sulfatide in SCN and tenascin-C on the cells surface of U-118MG cells.

Our previous pharmacokinetic studies of the SLC-DOX in healthy SD rats revealed that the clearance rate of free DOX is 1.93-fold higher than that of SCN-DOX [35]. The same improvement was found in the area under the plasma concentration-time curve from time 0 to infinity and apparent volume of distribution of free DOX in the current study (Table 3), indicating prolonged circulation time, slow clearance rate and enhanced bioavailability of SCN-DOX. In addition, there has been a significant improvement of biodistribution profile. As a widely used and efficient antitumor drug, however, DOX has a severe cardiotoxicity that limits its clinic utility [51]. Encapsulation of DOX into SCN resulted in a ∼4-fold lower DOX concentration in the heart of rats receiving SCN-DOX (Figure 4), which represents a significant improvement over those reported by others showing an approximately 1.5 times lower DOX accumulation in the heart with other liposomal DOX formulation compared with free DOX [18], [19]. Therefore, the significant reduction in accumulation of DOX in the heart indicates the potential of SCN-DOX in reducing the cardiotoxicity of DOX. This has been reinforced by our biochemical studies of the serum creatine kinase activity, which is a toxicologic indicator of severe cardiotoxicity [52] as well as cardiac troponin, another biomarker used for the detection and prevention of cardiotoxicity at an earlier phase [53]. Previous study by others revealed an increase of serum troponin level from week 10 after the first administration of DOX in Wistar rats [24]. Our present study revealed no discernable increase in serum troponin level in mice treated with SCN-DOX even 14 weeks after the onset of treatment, suggesting a remarkable reduction in cardiotoxicity of DOX delivered via SCN. Moreover, it is important to note that despite that SCN-DOX resulted in a high accumulation of DOX in the liver, better liver function than that in the animals received free DOX was observed at the end of the study, as demonstrated by a significant reduction of serum AST in mice treated with SCN-DOX (Figure 8).Of note, DOX is excreted predominantly through the hepatobiliary route [54] and there is a good negative correlation between serum AST activity and hepatic intrinsic clearance [55]. Therefore, it is tempting to speculate that SCN-DOX accumulated in the liver was not significantly taken up the hepatocytes but rather secreted via the bile duct. Further studies are under way to explore the mechanism underlying the reduced hepatotoxicity afforded by the SCN-DOX.

Although encapsulation with liposomes has been successful in overcoming cardiotoxicity and myelosuppression (for free DOX), the toxicity of liposomal DOX has shifted to the cutaneous toxicity [56]. Palmar-plantar erythrodysesthesia (PPE), also called hand-foot syndrome, is a toxic reaction associated with high accumulation of cytotoxic chemotherapeutics, including pegylated liposomal doxorubicin formulation in the skin [57]. High liposome localization in the skin has been reported previously in both nude mice and human [58]. Of particular interest is the fact that, although our SCN increased DOX delivery to the tumor and decreased the DOX accumulation in the heart, no increased delivery to the skin was observed. On the contrary, we observed a significantly reduced concentration of DOX in the skin of the mice treated with SCN-DOX repeatedly compared to the free DOX group (Figure 5), suggesting that SCN formulation of DOX could help to reduce the dose-limiting cutaneous toxicity displayed by other liposomal formulations of DOX.

On the other hand, the uptake of DOX in liver and spleen, which are tissues rich in cells of the reticuloendothelial system (RES), was higher for SCN-DOX when compared to free DOX (Figure 4), in agreement with previous studies [18], [59]. The increased accumulation of SCN-DOX in the organs of RES might be related to the particle size of our SCN [60], [61]. As studies indicated that conjugating polyethylene glycol (PEG) onto nanoliposomes can minimise the RES upkate of nanoliposomes [62], [63]. Further work is needed to reduce the uptake of SCN-DOX by RES. Of note, the measured concentration of DOX and SCN-DOX in various tissues was higher in the glioma-bearing mice than that in colon cancer xenograft study reported by us earlier [35]. This difference could largely be attributed to the fact that in the current study, the biodistribution study in tumor-bearing mice was performed after 6 injections of 5 mg/kg of DOX or SCN-DOX over a 6 week period while in our previous study the biodistribution study was performed after a single injection [35].Repeated injections of liposomal formulation are known to alter the PK of therapeutic agents [64]. Thus, the difference in tissue concentrations of DOX and SCN-DOX between animals receiving a single injection [35] and multiple injections (this study) might be resulted from the profoundly altered pharmacokinetic behaviour of the same agent after multiple administrations.

In addition to a benefit of reduced toxicity by SCN-DOX formulation, better treatment efficacy from animals treated with SCN-DOX over free DOX group were found in the tumor-bearing mice (Figure 6 and 7). Significantly higher concentrations of DOX in tumor from SCN-DOX treated mice were found after the repeated injections (Figure 5). Administration of SCN-DOX had superior tumor inhibitory effects compared to that of free DOX (Figure 6), manifested as both the inhibition of tumor growth and the increased life span (Figure 7).

Nano-carriers can be used to improve the treatment efficacy and reduce the side effects of drugs they encapsulated. Due to its high surface-to-volume ratio, functionalizing the surface of nanoparticles with ligands such as antibodies, aptamers, peptides, or small molecules that are tumor-specific or tumor-associated can promote the active binding of nanoparticles to tumors [1]. The composition and structure of the extracellular matrix in tumors are different from that in the normal tissues [65]. Certain extracellular matrix glycoproteins are highly up-regulated in many different cancers, including gliomas, breast cancer and ovarian cancer [66], [67]. Tenascin-C is a protein expressed at low levels in normal adult tissues but high levels in many tumors [68], including gilomas. Therefore, tenascin-C has been implicated as an important target for the treatment of cancer [69]. In our previous study, the key component of SCN, sulfatide, was demonstrated to mediate the binding and endocytic uptake of SCN in tumor cells via the interaction with tenascin-C [12]. It is tempting to speculate that sulfatide might be responsible for the observed improved therapeutic activity of SCN-DOX in tenascin-C expressing tumor model (U-118MG) used here. Further biochemical and molecular cell biological investigations will shed light on the mechanisms underlying the efficacy of this nanoliposome system and may thus reveal additional therapeutic strategies for treating tenascin-C positive tumors.
